# Development and Validation of the Digestive Function Assessment Instrument for Traditional Korean Medicine: Sasang Digestive Function Inventory 

**DOI:** 10.1155/2013/263752

**Published:** 2013-09-25

**Authors:** Misuk Lee, Na Young Bae, Minwoo Hwang, Han Chae

**Affiliations:** ^1^Department of Sasang Constitutional Medicine, Pusan National University Korean Medicine Hospital, Busan 626-789, Republic of Korea; ^2^Sasang and Personalized Medicine Research Center, Pusan National University, Busan 609-735, Republic of Korea; ^3^Division of Clinical Medicine, School of Korean Medicine, Pusan National University, Busan 626-870, Republic of Korea; ^4^Department of Sasang Constitutional Medicine, College of Korean Medicine, Kyung Hee University, Seoul 130-701, Republic of Korea; ^5^Division of Longevity and Biofunctional Medicine, School of Korean Medicine, Pusan National University, Busan 609-735, Republic of Korea; ^6^Department of Psychiatry, School of Medicine, Washington University, Saint Louis, MO 63110, USA

## Abstract

*Objective*. This study was conducted for development and validation of the Sasang Digestive Function Inventory (SDFI) with type-specific digestive function-related symptoms for identification of Sasang type and pathological pattern. *Methods and Materials*. We selected questionnaire items for pathophysiological symptoms using internal consistency analysis and examined construct validity using 193 healthy participants. Test-retest reliability with a four-week interval as well as convergent validity was examined using the Nepean Dyspepsia Index-Korean (NDIK), Functional Dyspepsia-Related Quality of Life (FDQOL), Dutch Eating Behavior Questionnaire (DEBQ), and Body Mass Index (BMI). *Results*. The 21-item SDFI showed satisfactory internal consistency (Cronbach's alpha = 0.743) and test-retest reliability (*r* = 0.886, *P* < 0.001). Three extracted subscales, SDFI-Digestion, SDFI-Appetite, and SDFI-Eating pattern, explained 56.02% of the total variance. The SDFI showed significant (*P* < 0.001) correlation with total symptom score of NDIK, FDRQOL-Eating status, DEBQ-External Eating scale, and BMI. The SDFI score of the Tae-Eum (50.62 ± 8.05) type was significantly (*P* < 0.001) larger than that of the So-Eum (43.11 ± 11.26) type. *Conclusion*. Current results demonstrated the reliability and validity of the SDFI and its subscales, which can be utilized as an objective instrument for diagnosis of Sasang types and assessment of the type-specific digestive function.

## 1. Introduction

Sasang typology is a traditional Korean personalized medicine incorporating medicinal herbs and acupuncture developed through Confucianism and clinical experience in Korea [1, 2]. It divides humans into four Sasang types: Tae-Yang (TY), So-Yang (SY), Tae-Eum (TE), and So-Eum (SE) which allows for distinctive disease diagnosis and intervention depending on their types. Therefore, studies on development and improvement of diagnostic methods were conducted using type-specific psychological [3–5], physical [2, 6, 7], and genetic [8, 9] characteristics as well as the type-specific morphological shapes of the face [7, 10] and body [11]. 

Although the morphological phenotype appears to be useful and objective, there has been more emphasis on clinical observations for pathophysiological symptoms and pathological pattern identification. The type-specific original symptoms (*素證*), that is, commonly observed Sasang type-specific pathophysiological symptoms regardless of health status, have been considered as pivotal information for diagnosis and intervention when the type identification based on the face or body shape could not provide confidence [12]. 

In Jae-ma Lee's book, Dong-Yi-Soo-Se-Bo-Won (*The Principle of Life Preservation in Oriental Medicine*), the original symptom is regarded as the most important clinical index [13, 14] in finalizing the Sasang type differentiation, pathological pattern identification, and the treatment itself.

*“*太陰少陰人體形或略相彷佛難辨疑似而觀其病證則必無不辨*, *人物形容仔細商量再三推移如有迷惑則參互病證明見無疑然後可以用藥最不可輕忽而一貼藥誤投重病險證一貼藥必殺人* (*東醫壽世保元*, *四象人辨證論*) *[1]*”*

*“Since the Tae-Eum and So-Eum Sasang type sometimes show similar morphological shapes on face and body parts, you should consider the pathological features for the Sasang type differentiation. If you are not sure about the Sasang type differentiation of patient, you should hold the medication until the confidence from the pathological features can be achieved. You should careful with the medication even with the simple and light disease, since a single dose can cause death in severe disease patient.” (Discussion on the Sasang type differentiation, The Principle of Life Preservation in Oriental Medicine).*

The Sasang type-specific original symptoms can be classified into five categories, including sleep [15–18], defecation [17, 19], urination [17], and sweating [20, 21], along with the most prominent and essential digestive function [12, 22–26]. However, sufficient studies on the pathophysiological symptoms with quantitative indexes were not implemented, and questionnaire items reflecting type-specific characteristics have been presented sporadically [12, 27].

In a previous study [12], we reviewed the pathophysiological characteristics of Sasang type-specific gastrointestinal symptoms and digestive functions and summarized the type-specific symptoms into three factors of digestion, appetite, and eating pattern. In addition, we also found the possibility of developing a more objective questionnaire using items collected from previous clinical studies [12, 22, 23, 27]. 

Therefore, based on previous clinical reviews, we constructed the theoretical framework of the Sasang Digestive Function Inventory (SDFI) [12, 27], as follows: the Tae-Eum type has higher digestive function, larger meal volume, faster eating speed, preference for fatty or salty food, more occurrences of gastritis, larger Body Mass Index, and frequent organic digestive malfunction when compared with the So-Eum type. The TE and SE types would have opposite characteristics in gastrointestinal and digestive functions. 

In this study, we developed the SDFI using questionnaire items from previous studies [12, 22, 23, 27] and examined its validity and reliability with the use of conventional digestion related questionnaires, such as Nepean Dyspepsia Index-Korean (NDIK) [28], Functional Dyspepsia-Related Quality of Life (FDQOL) [29], and Dutch Eating Behavior Questionnaire (DEBQ) [30], along with the Body Mass Index (BMI) [2]. 

The NDIK and FDQOL were widely used for examination of symptoms and quality of life changes in functional gastric diseases, and the DEBQ was commonly used for evaluation of dietary behaviors. The BMI is a numerical and physical index used to indicate digestive power and is frequently used for screening obesity. We examined the correlation of SDFI and these measures for the convergent validity and also presented significant differences between Sasang types in these digestion-related pathophysiological characteristics. 

This study would provide a foundation for measurement of Sasang type-specific multifaceted clinical symptoms and make possible mechanism studies on the type-specific pathophysiological symptoms. In addition, the Sasang typology inventories with acceptable validity and reliability would make the collaborative studies between Western and Eastern medicine researchers on the personalized medicine available. 

## 2. Method and Materials

### 2.1. Participants and Procedures

Participants in this study were 193 students from the School of Korean Medicine at Pusan National University, who gave written consent for conduct of the assessments. This study was approved by the Institutional Review Board (IRB) of the Pusan National University Korean Medicine Hospital (PNUKH-IRB-2012002). 

We administered the Questionnaire for Sasang Constitution Classification II (QSCCII) for identification of Sasang type while NDIK, FDQOL, DEBQ, BMI, and SDFI were examined for development and validation of the SDFI and its subscales. A total of 97 subjects completed the SDFI again for measurement of the test-retest reliability of the SDFI.

### 2.2. Measurements

#### 2.2.1. Questionnaire for Sasang Constitution Classification II (QSCCII)

The QSCCII, a Sasang typology-based inventory, is composed of 121 forced-choice items, including typical temperaments, body shapes, life style, and common health problems of each Sasang type. This questionnaire was developed in 1993 and revised in 1996 and has been used as an objective diagnostic tool in many studies examining the type-specific characteristics of Sasang typology [2]. The correctly predicted percentage of QSCCII was reported as 70.08% [31], and the internal consistency [32] measured using Cronbach's alpha was as follows: TY type was 0.57, SY type was 0.58, TE type was 0.59, and SE type was 0.63.

#### 2.2.2. Nepean Dyspepsia Index-Korean (NDIK)

The NDI is a reliable and validated disease-specific index for uninvestigated dyspepsia and functional dyspepsia, which measures symptoms and health-related quality of life. It was developed in 1999 [33], and the Korean version used in this study (NDIK) was standardized and validated in 2003 [28]. The internal consistency measured using Cronbach's alpha was 0.89–0.97, and the test-retest reliability was 0.86–0.96 [28]. 

The NDIK is composed of a symptom checklist and a disease-specific quality of life measure. In the current study, we used the symptom checklist, which includes 15 items rating common upper gastrointestinal symptoms using a 5- or 6-point Likert scale. The higher score indicates higher severity of symptoms.

#### 2.2.3. Functional Dyspepsia-Related Quality of Life (FDQOL)

The FDQOL measures the quality of life of functional dyspepsia patients with the reference of Health-Related Quality of Life in 2006, and its internal consistency measured with Cronbach's alpha was 0.94 [29]. The FDQOL was composed of 21 items with a 5-point Likert scale, and a higher score indicates lower quality of life. It is composed of four subscales, including eating status (FDQOL-E), liveliness status (FDQOL-L), psychological status (FDQOL-Psy), and role-functioning status (FDQOL-R). 

#### 2.2.4. Dutch Eating Behavior Questionnaire (DEBQ)

The DEBQ is a self-report screening instrument that measures patterns of eating behaviors [34]. The DEBQ has three subscales measuring Restrained Eating (DEBQ-R, 10 items), Emotional Eating (DEBQ-EM, 13 items), and External Eating (DEBQ-EX, 10 items) with a 5-point Likert scale. The Korean version was standardized and validated in 1996, and its Cronbach's alpha for the Restrained Eating, Emotional Eating, and External Eating scales was reported as 0.90, 0.93, and 0.79, respectively [30]. 

#### 2.2.5. Body Mass Index (BMI)

Body Mass Index (BMI) is a widely used measure for body shape and body fat mass with height and weight. It is calculated as weight (kg) divided by height (m) squared (2). 

### 2.3. Sasang Digestive Function Inventory (SDFI) Development

The previous systematic review [12] showed that Sasang type-specific pathophysiological symptoms in the gastrointestinal tract can be categorized as three factors, including digestion, appetite, and eating patterns, and the TE and SE types showed distinctively opposite clinical features.

We conducted an extensive review of previous articles and selected 33 items which are supposed to represent type-specific characteristics in clinical settings [12, 22, 23, 27]. After examining and editing the items to be understood easily by the patients, pilot study with twenty subjects was made to get the preliminary 31 items. All subjects in the pilot study were asked to complete the questionnaires and were subsequently interviewed for identification of any difficulties with understanding the questionnaire items. Each item is measured using a 5-point Likert scale (0 = not at all to 4 = very true), and a few items were calculated in reverse. SDFI and its subscales were calculated after factor extraction and item selection. 

### 2.4. Statistical Analysis

#### 2.4.1. Explorative Factor Analysis

The 31 preliminary items were subjected to explorative factor analysis for examination of the possible structure of the SDFI using Principle Component Analysis and Varimax rotation with an Eigenvalue over 1.0 as the criteria. We also performed Parallel Analysis in order to obtain the right factor numbers using Monte Carlo PCA for Parallel Analysis, which estimates an average distribution of eigenvalues based on a random process that can be compared with the calculated distribution [35, 36]. The meaning of extracted factors was analyzed by review of questionnaire items while referring to the factor loadings and the initial structure design.

#### 2.4.2. Test-Retest Reliability

The test-retest data for the reliability examination over a period of four weeks was analyzed using correlational analysis. The reliability of the SDFI and its subscales was determined using Pearson's correlational coefficients with 97 subjects.

#### 2.4.3. Convergent Validity

The convergent validity of the SDFI and its subscales was examined in order to confirm the construct of Sasang type-specific digestive functions by comparing the relationship with the well-established NDIK, FDQOL, DEBQ, and BMI, which measure digestive function and its subsequent physical characteristics. We used Pearson's correlational coefficients exceeding the minimum acceptable value of 0.3.

#### 2.4.4. SDFI and Sasang Typology

Demographic differences between Sasang-type groups were tested using ANOVA for continuous variables (age and BMI) and chi-square test for categorical variables (gender). The SDFI and its subscales, NDIK, FDQOL DEBQ, and BMI, were subjected to Analysis of Variance (ANOVA) for examination of the differences between Sasang type groups and Scheffe or Dunnett's T3 was used for post hoc analysis. 

The data are presented as means and standard deviations or frequency with percentage. All analyses were performed using PASW Statistics 18.0 for Windows (IBM, Armonk, NY, USA) and *P* values of 0.05, 0.01, and 0.001 were used for significance.

## 3. Results

### 3.1. Characteristics of the Participants

Since fifteen participants did not receive their Sasang type classification based on the Questionnaire for Sasang Constitution Classification II (QSCCII), SDFI, and NDIK, FDQOL, DEBQ, and BMI were examined with the remaining 178 participants (82 males and 96 females; mean age 29.12 ± 4.97; range 22–45). 

The mean age of each Sasang type was 29.49 ± 4.83 for the TE type (29 males and 16 females), 29.67 ± 5.13 for the SY type (17 males and 29 females), and 28.63 ± 4.96 for the SE type (36 males and 51 females). Significant differences in gender distribution were observed between Sasang type groups (*χ* ^2^ = 8.423, df = 2, *P* = 0.015), while no significant (*F* = 0.830, df = 2,174, *P* = 0.438) differences in age were observed between Sasang-type groups (Table 5). 

### 3.2. Analysis of SDFI and Its Subscale

#### 3.2.1. Factor Extraction and Item Selection

After examining the internal consistency and the context, we deleted two items from the 31 preliminary items due either to low Cronbach's alpha or duplicated meanings. The Varimax rotation procedure, with 29 items, yielded three factors. We excluded eight items because four items had factor loading lower than 0.5, two items had a factor loading difference lower than 0.1, and two items were classified as other factors initially designed. 

As expected from the previous systematic review, the final SDFI, with 21 items (Table 1), ultimately confirmed three interpretable subscales. The SDFI showed internal consistency of 0.743, and the SDFI items showed a Cronbach's alpha of 0.716–0.774. 

The SDFI showed a cumulative explanatory value of 56.02% of the variance (Table 2). The first factor, which accounted for 25.88% of the variance, was named SDFI-Digestion (SDFI-D) and measured the status of digestive function and upper gastrointestinal symptoms (Table 1). Factor 2, which accounted for 15.55% of the variance, was named SDFI-Appetite (SDFI-A) and measured the degree of appetite and its variation according to mood or physical condition. Factor 3, which accounted for 14.584% of the variance, was named SDFI-Eating habit (SDFI-E) and measured regularity and size of the meal and the speed of eating. 

Cronbach's alpha of SDFI-D, SDFI-A, and SDFI-E was 0.784, 0.798, and 0.757, respectively (Table 2). Furthermore, the SDFI showed significant correlation with SDFI-D (*r* = 0.797, *P* < 0.001), SDFI-A (*r* = 0.658, *P* < 0.001), and SDFI-E (*r* = 0.525, *P* < 0.001). SDFI-A showed significant correlation with SDFI-E (*r* = 0.379, *P* < 0.001), while the SDFI-D did not show significant correlation with other subscales (Table 4).

#### 3.2.2. Test-Retest Reliability

The test-retest reliability of the SDFI and its subscales over four weeks was analyzed using Pearson's correlational analysis. Overall test-retest reliability was found to be 0.886, and reliability of the three subscales of SDFI-D, SDFI-A, and SDFI-E was 0.859, 0.801, and 0.909, respectively (Table 3).

#### 3.2.3. Convergent Validity of SDFI with NDIK, FDQOL, DEBQ, and BMI

The convergent validity of the SDFI was examined with the NDIK, FDQOL DEBQ, and BMI using Pearson's correlation analysis (Table 4). SDFI showed significant correlation with NDIK (*r* = −0.431, *P* < 0.001), FDQOL-E (*r* = −0.391, *P* < 0.001), DEBQ-EX (*r* = 0.301, *P* < 0.001), and BMI (*r* = 0.299, *P* < 0.001). 

SDFI-D showed significant correlation with NDIK (*r* = −0.585, *P* < 0.001), FDQOL (*r* = −0.433, *P* < 0.001), FDQOL-E (*r* = −0.430, *P* < 0.001), FDQOL-L (*r* = −0.339, *P* < 0.001), and FDQOL-Psy (*r* = −0.364, *P* < 0.001). SDFI-A showed significant correlation with DEBQ-EX (*r* = 0.483, *P* < 0.001). SDFI-E showed significant correlation with DEBQ (*r* = 0.481, *P* < 0.001), DEBQ-EM (*r* = 0.441, *P* < 0.001), DEBQ-EX (*r* = 0.493, *P* < 0.001), and BMI (*r* = 0.310, *P* < 0.001) (Table 4).

### 3.3. SDFI, NDIK, FDQOL, DEBQ, and BMI of Each Sasang Type

The pathophysiological features in digestive functions were measured using NDIK, FDQOL, DEBQ, and BMI in order to determine whether the SDFI showed significant differences between Sasang type groups (Table 5) and to determine whether the measures replicate previous clinical findings [12]. 

Using ANOVA, significant differences in the SDFI (*F* = 8.286, df = 2,175, *P* < 0.001), SDFI-D (*F* = 5.102, df = 2,175, *P* = 0.007), and SDFI-E (*F* = 5.352, df = 2,175, *P* = 0.006) were observed between Sasang type groups. Post hoc analysis showed that the TE type (50.62 ± 8.05, 25.22 ± 5.55, 9.89 ± 3.38) was significantly (*P* < 0.001, *P* < 0.001 and *P* = 0.006) higher than the SE type (43.11 ± 11.26, 20.98 ± 7.67, 7.66 ± 3.96) in SDFI, SDFI-D, and SDFI-E, respectively (Table 5). 

There was significant difference with FDQOL (*F* = 3.225, df = 2,175, *P* = 0.042) between Sasang type groups, and the post hoc analysis showed that the TE type (12.31 ± 9.49) was significantly (*P* = 0.011) lower than the SE type (18.31 ± 13.42). 

The significant difference on the DEBQ-R (*F* = 3.135, df = 2,175, *P* = 0.046) between Sasang type groups was demonstrated with ANOVA. Post hoc analysis showed that the DEBQ-R of the TE type (27.62 ± 6.35) was significantly (*P* = 0.046) higher than that of the SE type (24.07 ± 8.21) (Table 5).

The significant difference on the BMI (*F* = 33.928, df = 2,171, *P* < 0.001) between Sasang type groups was demonstrated with ANOVA. Post hoc analysis showed that the BMI of the TE type (24.71 ± 3.07) was significantly (*P* < 0.001) lower than that of the SY type (21.94 ± 2.26) and the SE type (20.75 ± 2.51) (Table 5).

## 4. Discussion and Conclusion

The Sasang typology is a traditional Korean personalized medicine focusing on type-specific diagnosis and intervention for safe and effective medical treatment, including acupuncture and medicinal herbs [1, 2]. In this study, in order to provide more objective identification of Sasang type and pathological pattern, we developed the Sasang Digestive Function Inventory (SDFI), which measures the digestion-related pathophysiological symptoms of Sasang typology, and validated its robust structure. 

The Reliability and validity examination of the 21-item SDFI demonstrated that the SDFI has three domains with acceptable internal consistency (Cronbach's alpha = 0.743) and test-retest reliability (*r* = 0.886, *P* < 0.001). The three subscales of the SDFI measure three facets of digestive functions, namely, SDFI-D (digestive function, 10 items), SDFI-A (appetite, six items), and SDFI-E (eating habits, five items), which was suggested by previous studies [12] on Sasang type-specific clinical features (Table 1). 

The SDFI and its subscales measure whether the subject has better digestive function and appetite, larger volume and higher speed of the meal, and higher occurrence of digestive disease than others (Table 1). As expected, score for the TE type was significantly higher than that of the SDFI, SDFI-D, and SDFI-E (Table 5); these results demonstrate that this questionnaire firmly measures Sasang type-specific digestive system-related pathophysiological symptoms, as predicted [12, 22, 23, 27].

We measured digestive function-related clinical features of each Sasang type using NDIK, FDQOL, and DEBQ and evaluated the usefulness of SDFI and its subscales by correlation analysis in clinical use as convergent validity (Table 4). The SDFI showed significant correlation with total symptom score of NDIK (*r* = −0.431, *P* < 0.001), FDRQOL-E (*r* = −0.391, *P* < 0.001), and DEBQ-EX (*r* = 0.301, *P* < 0.001). 

As for the SDFI subscales, the SDFI-D measures digestive functions and upper gastrointestinal symptoms and showed significant correlation coefficients with NDIK (*r* = 0.59) and FDQOL (*r* = −0.43). The NDIK [33] examines the frequency and degree of 15 gastrointestinal symptoms in functional digestive disease, and the FDQOL [29] measures the change of quality of life by functional digestive diseases. Although the NDIK and FDQOL deal with patients rather than healthy subjects of the SDFI, the purpose of the instrument is assessment of the digestive function and its influence on quality of life, and this makes the use of NDIK and FDQOL for convergent validity suitable. 

The SDFI-A and SDFI-E measure the pattern of eating, such as appetite, volume, and speed of the meal, and these two subscales have significant correlation coefficients (*r* = 0.379, *P* < 0.001). The SDFI-A showed significant correlation with DEBQ-EX (*r* = 0.48), and the SDFI-E showed significant correlation with DEBQ, DEBQ-EM, and DEBQ-EX as 0.48, 0.44, and 0.49, respectively. The DEBQ [30] measures the reason and type of overeating or binge eating with respect to emotional, external, and restrained psychological reasons. These results demonstrate that the SDFI-A and SDI-E measure behavioral characteristics of Sasang type-specific eating. 

In this study, BMI was used as a representative physical measure indicating digestive power and obesity. BMI showed significant correlation with SDFI (*r* = 0.299) and SDFI-E (*r* = 0.310), and BMI of the TE type was significantly higher than that of other Sasang types, which was repeatedly reported in previous studies [2, 6, 7]. These results demonstrate that the higher speed and larger volume of meals with TE type result in higher calorie intake for higher BMI, obesity, and metabolic syndrome [37]. As for the clinical studies, BMI showed negative correlation with functional dyspepsia [38], nonulcerative dyspepsia [39], and gastroptosis [40], which was not frequent with the TE type [12]. 

We analyzed the significant differences between Sasang types in NDIK, FDQOL, and DEBQ to determine the characteristics of the TE Sasang type groups compared with others. The TE type showed a low NDIK score, which measures the frequency of upper gastrointestinal symptoms. The TE also showed a significantly lower score than the SE type on FDQOL, which measures how much the quality of life was worsened by digestive disease. The TE type showed a significantly higher DEBQ-R score, which represents periodic overeating due to the suppressed desire for the food itself. These results are similar to those of previous clinical studies on Sasang type-specific gastrointestinal features [12, 22, 23, 27]. 

In this study, we developed the SDFI as a clinical index for measurement of Sasang type-specific pathophysiological symptoms, which are regarded as the most important clinical features in Sasang typology practice. Objective measures of the type-specific symptoms would be useful for making Sasang type differentiation and type-specific symptom based-disease diagnosis and intervention more reliable. 

For example, the location of the pathogen and severity of disease can be identified with the status of digestion in the SE and SY types [41, 42] and with the abdominal pain and meal volume in the TE type [43]. If objective instruments for type-specific clinical symptoms, including defection/urination [17, 19], sweating [20, 21], and sleep [15, 16], could be developed, safer and more effective clinical practice with acupuncture and medicinal herbs could be achievable. 

In previous studies, the Sasang Personality Questionnaire (SPQ) [3] showed SE-TE-SY dimension for psychosocial traits, and BMI [2] showed SE-SY-TE dimension for physical traits. We also confirmed here the SE-SY-TE dimension for digestive system related clinical symptoms. More effective identification of Sasang type and disease pattern can be achieved if we can use several multifacet indexes all together for development of profiles of characteristics related to Sasang typology and/or disease patterns [5]. 

Study considering the relation between these indexes would also be a foundation for innovative clinical studies in traditional medicine. A close relationship of gastrointestinal problems with psychosocial factors, including depression, anxiety, and stress, has recently been reported [44]. The SE type, which denotes a low score for both SPQ and SDFI, is psychologically introvert and neurotic [3] and has physically small BMI and lower digestive power. These correlations in SE type can be regarded as groundwork for the study of SE type-specific pathophysiological characteristics and mechanism of action in type-specific acupuncture and medicinal herbs. The Ginseng Radix, Glycyrrhizae Radix, and Cinnamomi Cortex are regarded as type-specific useful medicinal herbs, and HT7, SP3, and LI4 are regarded as type-specific acupuncture points for the SE Sasang type with higher efficacy and safety [3, 45].

This study has several limitations that may affect generalizability of the results. First, the subjects were relatively young, healthy persons, and this may dampen the influence of living habits and show minor chronic digestive symptoms. Conduct of studies using clinical samples with gastrointestinal disease, metabolic syndrome [37], diabetes mellitus [46], and others is needed in order to confirm the difference in SDFI score between Sasang type groups. Studies with consideration for age and gender differences in Sasang typology should also be conducted if the SDFI is to be used as a generalized instrument for measurement of Sasang type-specific digestive function.

 Second, because the SDFI and other instruments used in this study are self-report questionnaire and may have the influence of response bias, the clinical validity should be confirmed again using other objective measures of gut hormones [47], triglyceride and cholesterol [2], body fat mass [2], insulin resistance [48], and blood pressure [49]. Last but not the least, clinical features of each Sasang type in this study using the QSCCII are a replication of previous clinical studies and reviews [12, 22–26]; there is still a possibility of socio-economical characteristics that may affect Sasang type classification method. 

The SDFI would be useful for the Sasang type and pattern identification since the SDFI has theoretical background, subscale construct, and questionnaire items based on an extensive review of previous clinical studies [12] and has shown statistical reliability and convergent validity as expected. In conclusion, the Sasang Digestive Function Inventory was developed with type-specific pathophysiological gastrointestinal symptoms and was validated using established western digestive-system-related instruments. With further clinical investigation, the SDFI would serve as an objective instrument for personalized medicine practice in traditional medicine with medical herbs and acupuncture. 

## Figures and Tables

**Table 1:** Extracted and rotated factor-loading matrix of SDFI subscales and questionnaire items.

	Questionnaire items	Factor loading		
		1	2	3
SDFI-D	(1) I usually tend to digest well compared to others.	−**0.724**	0.302	0.200
	(2) I often suffered from digestion problems since I was young.	**0.700**	−0.029	−0.157
	(3) I tend to easily get an upset stomach.	**0.748**	−0.032	−0.074
	(4) After consuming milk, food made of flour, cold food, or greasy food, I tend to feel uncomfortable. I cannot digest well and my stomach gets filled with gas quickly.	**0.737**	0.036	−0.022
	(5) I cannot digest well when I am in a sensitive mood.	**0.776**	0.028	0.007
	(6) I cannot digest well when my body is in bad condition.	**0.785**	−0.009	−0.034
	(7) I have frequently experienced pressure or bloat in upper abdomen.	**0.757**	−0.046	0.210
	(8) I have frequently experienced discomfort or burn in upper abdomen.	**0.697**	−0.058	0.202
	(9) I often feel bloated or full without eating much.	**0.664**	−0.112	0.271
	(10) I tend to get full quickly after eating a little.	**0.561**	−0.215	−0.132

SDFI-A	(1) I wait feeling hungry around the meal time.	0.033	**0.781**	0.087
	(2) I enjoy the meal during the meal.	0.028	**0.812**	0.166
	(3) I feel pleasant after eating a meal.	−0.285	**0.652**	0.188
	(4) I like food and enjoy eating.	0.016	**0.727**	0.390
	(5) I like to try even new food that I had never tried or seen before.	−0.191	**0.596**	0.266
	(6) I usually eat regularly at certain time of the day.	−0.094	**0.553**	−0.207

SDFI-E	(1) I eat more than usual when I am under stress or when I'm in a bad mood.	0.153	0.233	**0.547**
	(2) I tend to eat more portion than others usually.	−0.151	0.199	**0.799**
	(3) I eat until I feel full when I'm having a meal.	0.082	0.326	**0.751**
	(4) I tend to overeat or binge more than 3 times a week.	0.157	0.082	**0.832**
	(5) I tend to finish a meal in less than 10 minutes.	−0.281	−0.116	**0.534**

Bold represents factor loading over 0.5.

SDFI: Sasang Digestive Function Inventory; SDFI-D: SDFI-Digestion; SDFI-A: SDFI-Appetite; SDFI-E: SDFI-Eating pattern.

**Table 2:** Extraction of SDFI subfactors with explorative factor analysis using Varimax rotation.

Factor	Extraction sums of squared loadings			Rotation sums of squared loadings		
	Eigenvalue	Percent of variance	Cumulative percent	Eigenvalue	Percent of variance	Cumulative percent
1	5.707	27.176	27.176	5.435	25.882	25.882
2	4.196	19.983	47.159	3.266	15.551	41.433
3	1.860	8.857	56.016	3.063	14.584	56.016

**Table 3:** Test-retest reliability for the SDFI and its subscales.

	Score of the test	Score of the retest	Pearson's correlation coefficient
SDFI	46.36 ± 9.93	46.27 ± 10.09	0.886***
SDFI-D	22.18 ± 7.68	22.12 ± 7.40	0.859***
SDFI-A	15.23 ± 3.49	15.11 ± 3.59	0.801***
SDFI-E	8.96 ± 3.99	9.03 ± 3.82	0.909***

*P < 0.05, **P < 0.01, ***P < 0.001.

SDFI: Sasang Digestive Function Inventory; SDFI-D: SDFI-Digestion; SDFI-A: SDFI-Appetite; SDFI-E: SDFI-Eating pattern.

**Table 4:** Correlation between SDFI, subscales of SDFI, NDIK, FDQOL, DEBQ, and BMI.

	SDFI			NDIK	FDQOL					DEBQ				BMI
	SDFI-D	SDFI-A	SDFI-E		FDQOL	FDQOL-E	FDQOL-L	FDQOL-Psy	FDQOL-R	DEBQ	DEBQ-R	DEBQ-EM	DEBQ-EX	
SDFI	0.797***	0.658*	0.525***	−0.431***	−0.245**	−0.391***	−0.135	−0.200**	−0.127	0.107	−0.032	0.037	0.301***	0.299***

SDFI-D		0.210*	0.029	−0.585***	−0.433***	−0.430***	−0.339***	−0.364***	0.298***	−0.249***	−0.141	−0.276***	−0.081	0.213**
SDFI-A			0.379***	−0.157*	−0.063	−0.212**	0.039	−0.043	−0.034	0.293***	0.048	0.194**	0.483***	0.110
SDFI-E				0.115	0.235**	−0.022	0.251***	0.204**	0.268***	0.481***	0.138	0.441***	0.493***	0.310***

*P < 0.05, **P < 0.01, ***P < 0.001.

Bold represents correlation coefficient over 0.3.

SDFI: Sasang Digestive Function Inventory; SDFI-D: SDFI-Digestion; SDFI-A: SDFI-Appetite; SDFI-E: SDFI-Eating pattern; NDIK: total symptom score of Nepean Dyspepsia Index-Korean; FDQOL: Functional Dyspepsia-Related Quality of Life; FDQOL-E: FDQOL-Eating status; FDQOL-L: FDQOL-Liveliness; FDQOL-Psy: FDQOL-Psychological status; FDQOL-R: FDQOL-Role-Functioning status; DEBQ: Dutch Eating Behavior Questionnaire; DEBQ-R: DEBQ-Restrained Eating scale; DEBQ-EM: DEBQ-Emotional Eating scale; DEBQ-EX: DEBQ-External Eating scale; BMI: Body Mass Index.

**Table 5:** Demographic features and SDFI, NDIK, FDQOL, DEBQ, and BMI scores of each Sasang type group.

	Tae-Eum	So-Yang	So-Eum	Statistical analysis
Sex* (male/female)	45 (29/16)	46 (17/29)	87 (36/51)	*χ* ^2^ = 8.423, df = 2, *P* = 0.015
Age	29.49 ± 4.83	29.67 ± 5.13	28.63 ± 4.96	
BMI***	24.71 ± 3.07	21.94 ± 2.26	20.75 ± 2.51	*F* = 33.928, df = 2,171, *P* < 0.001 TE > SY***, TE > SE***

SDFI***	50.62 ± 8.05	46.78 ± 9.81	43.11 ± 11.26	*F* = 8.286, df = 2,175, *P* < 0.001 TE > SE***
SDFI-D**	25.22 ± 5.55	23.00 ± 8.09	20.98 ± 7.67	*F* = 5.102, df = 2,175, *P* = 0.007 TE > SE**
SDFI-A	15.51 ± 3.09	15.13 ± 3.79	14.48 ± 4.30	
SDFI-E**	9.89 ± 3.38	8.65 ± 3.65	7.66 ± 3.96	*F* = 5.352, df = 2,175, *P* = 0.006 TE > SE**

NDIK	16.13 ± 13.52	23.87 ± 21.24	23.44 ± 21.94	

FDQOL*	12.31 ± 9.49	16.93 ± 14.85	18.31 ± 13.42	*F* = 3.225, df = 2,175, *P* = 0.042 TE < SE*
FDQOL-E	1.24 ± 2.22	2.26 ± 3.61	2.18 ± 3.12	
FDQOL-L	6.18 ± 4.10	7.26 ± 4.80	7.54 ± 4.12	
FDQOL-Psy	2.47 ± 2.83	4.13 ± 4.90	4.48 ± 5.06	
FDQOL-R	2.42 ± 3.19	3.28 ± 4.28	4.10 ± 4.54	

DEBQ	84.00 ± 16.17	83.09 ± 16.93	82.83 ± 19.47	
DEBQ-R*	27.62 ± 6.35	25.46 ± 8.03	24.07 ± 8.21	*F* = 3.135, df = 2,175, *P* = 0.046 TE > SE*
DEBQ-EM	26.47 ± 9.70	27.00 ± 9.96	28.25 ± 11.21	
DEBQ-EX	29.91 ± 5.32	30.63 ± 6.64	30.51 ± 5.77	

*P < 0.05, **P < 0.01, ***P < 0.001.

SDFI: Sasang Digestive Function Inventory; SDFI-D: SDFI-Digestion; SDFI-A: SDFI-Appetite; SDFI-E: SDFI-Eating pattern; NDIK: total symptom score of Nepean Dyspepsia Index-Korean; FDQOL: Functional Dyspepsia-Related Quality of Life; FDQOL-E: FDQOL-Eating status; FDQOL-L: FDQOL-Liveliness; FDQOL-Psy: FDQOL-Psychological status; FDQOL-R: FDQOL-Role-Functioning status; DEBQ: Dutch Eating Behavior Questionnaire; DEBQ-R: DEBQ-Restrained Eating scale; DEBQ-EM: DEBQ-Emotional Eating scale; DEBQ-EX: DEBQ-External Eating scale; BMI: Body Mass Index.
